# Clara cell protein in bronchoalveolar lavage fluid: a predictor of ventilator-associated pneumonia?

**DOI:** 10.1186/cc9418

**Published:** 2011-01-11

**Authors:** Marijke J Vanspauwen, Catharina FM Linssen, Cathrien A Bruggeman, Jan A Jacobs, Marjolein Drent, Dennis CJJ Bergmans, Walther NKA van Mook

**Affiliations:** 1 Department of Medical Microbiology, CAPHRI School, Maastricht University Medical Centre, P. Debyelaan, Maastricht NL-6229HX, the Netherlands; 2 Department of Respiratory Medicine and Ild Care Team, Maastricht University Medical Centre, P. Debyelaan, Maastricht NL-6229HX, the Netherlands; 3 Department of Intensive Care Medicine, Maastricht University Medical Centre, P. Debyelaan, Maastricht NL-6229HX, the Netherlands; 4 Department of Clinical Sciences, Prins Leopold Institute of Tropical Medicine, Nationalestraat, Antwerp B-2000, Belgium

## Abstract

**Introduction:**

Clara cell protein 10 (CC-10) has been associated with inflammatory and infectious pulmonary diseases. This study evaluates CC-10 concentrations in bronchoalveolar lavage (BAL) fluid as a potential marker of ventilator-associated pneumonia (VAP).

**Methods:**

Between January 2003 and December 2007, BAL fluid samples obtained from critically ill patients at the intensive care unit of the Maastricht University Medical Centre clinically suspected of having VAP were included. Patients were divided into two groups: (1) microbiologically confirmed VAP (the VAP group) and (2) microbiologically unconfirmed VAP (the non-VAP group). The concentration of CC-10 was measured by means of a commercially available enzyme-linked immunosorbent assay kit, and retrospective analysis was performed. Areas under the curve of receiver operating characteristic curves were calculated for CC-10 concentrations.

**Results:**

A total of 196 patients (122 men, 74 women) were included. A total of 79 (40%) of 196 cases of suspected VAP were microbiologically confirmed. The median CC-10 concentration in the VAP group was 3,019 ng/mL (range, 282 to 65,546 ng/mL) versus 2,504 ng/mL (range, 62 to 30,240 ng/mL) in the non-VAP group (*P *= 0.03). There was no significant difference in CC-10 concentrations between patients treated with or without corticosteroids (*P *= 0.26) or antibiotic therapy (*P *= 0.9). The CC-10 concentration did not differ significantly between patients with Gram-positive versus Gram-negative bacteria that caused the VAP (*P *= 0.06). However, CC-10 concentrations did differ significantly between the late-onset VAP group and the non-VAP group.

**Conclusions:**

The CC-10 concentration in BAL fluid yielded low diagnostic accuracy in confirming the presence of VAP.

## Introduction

Clara cell protein 10 (CC-10) is a low-molecular-weight protein secreted into the alveoli in large quantities by nonciliated Clara cells [1,2]. CC-10 has structural homology with rabbit uteroglobin, which has immunosuppressive, anti-inflammatory, antiprotease and anti-phospholipase A_2 _activities [1,3,4]. This profile suggests a possible anti-inflammatory role for human CC-10 [4]. In line with these findings, differences in serum CC-10 concentrations have been demonstrated in several inflammatory lung diseases. Bronchial asthma and chronic eosinophilic pneumonia (CEP) have been associated with decreased serum CC-10, while patients with idiopathic interstitial pneumonia (IIP) demonstrated increased levels of CC-10 in serum and bronchoalveolar lavage (BAL) fluid [4]. Moreover, some studies in which pulmonary infectious diseases were investigated have suggested that CC-10 activity is influenced by the type of microorganism which is isolated. *Pseudomonas aeruginosa *has been shown to decrease CC-10 promoter activity, leading to a decrease in CC-10 mRNA and eventually to a decrease in the concentration of CC-10 [5,6]. The microscopic examination of BAL fluid is appreciated for various clinical applications. It is routinely used in the assessment of interstitial lung diseases, suspected cases of ventilator-associated pneumonia (VAP) and opportunistic lung infections [7-10]. VAP frequently develops in patients who are on mechanical ventilation in the intensive care unit (ICU) and is associated with high costs, morbidity and mortality, especially when treatment is delayed [11,12]. Microorganisms frequently associated with VAP are *S. aureus*, *P. aeruginosa *and the Enterobacteriaceae [13]. Unfortunately, the differentiation between VAP and noninfectious respiratory conditions mimicking VAP is difficult, and the culture of BAL fluid takes up to 48 hours. Microscopic examination of BAL fluid can be helpful in distinguishing VAP from noninfectious conditions mimicking VAP [14]. The differential cell count, especially the percentage of cells with intracellular organisms (ICOs), can be helpful in the diagnosis of VAP [14]. Furthermore, the percentage of ICOs is not influenced by antibiotic therapy in the 72 hours prior to the BAL. This makes it an important parameter for distinguishing VAP from non-VAP conditions [15]. However, BAL fluid workup and its microscopic analysis are time-consuming and must be done by experienced technicians. Therefore, different biological markers (for example, soluble triggering receptor expressed on myeloid cells (sTREM-1), procalcitonin, C-reactive protein) have been proposed as candidates for a rapid diagnostic test for VAP, but all failed to sufficiently discriminate VAP from other respiratory conditions mimicking VAP [16-20]. Procalcitonin, C-reactive protein and sTREM-1 were previously investigated by our group in the same patient population as the one in the present study. However, these markers could not accurately distinguish VAP from other respiratory conditions mimicking VAP [16,17]. Because of the possible anti-inflammatory role of CC-10, we hypothesise that CC-10 concentrations may be increased in patients with VAP. Therefore, the present study was designed to evaluate CC-10 in BAL fluid as a potential marker of VAP in critically ill patients in whom VAP is suspected.

## Materials and methods

### Sampling technique

This study was performed at the 17-bed general ICU of the University Hospital Maastricht (Maastricht, the Netherlands). During a 59-month period (January 2003 to December 2007), we considered consecutive BAL fluid samples obtained from patients who had undergone mechanical ventilation for more than 48 hours and were clinically suspected of having pneumonia. Only the first episode of VAP was included. Clinical suspicion of VAP was based on the criteria described by Bonten *et al.*[8] (Table 1). Bronchoscopies with BAL were performed as previously described [21,22]. In short, chest X-rays were performed to identify the affected lung segment. In those cases in which the affected segment could not be reached and in cases of patients with general opacification, the lingula was sampled. Bronchoscopies and subsequent lavage were performed prior to new antibiotic treatment and by experienced pulmonary physicians. Four fractions of 50 mL each of sterile saline (0.9% NaCl at room temperature) were instilled into the affected subsegmental bronchus and immediately aspirated and recovered. The BAL fluid samples were transported to the laboratory within 15 minutes of collection and were analysed immediately upon arrival in the laboratory.

**Table 1:** Criteria for clinical suspicion of ventilator-associated pneumonia^a^

Criteria
I. At least three positive results of the following four criteria
1. Rectal temperature > 38°C or < 35.5°C
2. Blood leucocytosis (> 10 × 10³/mm³) and/or left shift of blood leucopenia (< 3 × 10³/mm³)
3. > 10 leukocytes per high-power magnification field in Gram stain of tracheal aspirate
4. Positive culture from tracheal aspirate
II. New, persistent progressive infiltrate visualised on chest radiograph

^a^The diagnosis of clinical suspicion of ventilator-associated pneumonia was made when criteria I and II were positive. The criteria listed here are as described by Bonten *et al.*[8].

### Laboratory processing

The first fraction of BAL fluid representing the bronchial fraction was not used in this study. The remaining three fractions (alveolar fractions) were pooled and processed as previously described [23,24]. The bronchoalveolar lavage fluid workup included total cell count, differential cell count and quantitative culture for bacteria and yeasts. On the basis of clinical suspicion, additional diagnostic tests were added, such as culture for filamentous fungi, *Mycobacteria *spp. and *Legionella *spp., as well as polymerase chain reactions for the detection of *Chlamydophyla pneumoniae*, *Mycoplasma pneumoniae *and viruses.

### Exclusion criteria

Bronchoalveolar lavage fluid samples were excluded if (1) the recovered volume was less than 20 mL; (2) the total cell count was less than 60,000 cells/mL; (3) excessive amounts of intercellular debris, red blood cells or damaged red blood cells were present; or (4) more than 1% squamous epithelial cells were present [17]. In a small percentage (< 5%) of patients suspected of having VAP, BAL could not be performed because of a high risk of severe complications and/or a high risk of death. Criteria for not performing a BAL are (1) fraction of inspired oxygen > 65% and (2) severe right-sided heart failure. A high level of positive end-expiratory pressure or a low thrombocyte count was not considered an exclusion criteria.

This study was approved by the institutional review board and the ethics committee of the Maastricht University Medical Centre, and informed consent was obtained from patients or their next of kin.

### Definition of confirmed ventilator-associated pneumonia

VAP was microbiologically confirmed if BAL fluid cultures yielded ≥ 10^4 ^colony-forming units (CFU)/mL and/or microscopic analysis revealed ≥ 2% intracellular organisms [17]. In the case of mixed infections, either (1) one single microorganism had to yield a concentration of ≥ 10^4 ^CFU/mL or (2) the sum of the different microorganisms had to be ≥ 10^4 ^CFU/mL. According to these criteria, patients were divided into two groups: (1) microbiologically confirmed VAP (the VAP group) and (2) microbiologically unconfirmed VAP (the non-VAP group). Early-onset VAP was defined as VAP occurring within 7 days after intubation, whilst late-onset VAP was defined as VAP occurring more than 7 days after intubation [13,25].

### Collection of clinical data

Collected data included patients' demographic characteristics, such as age and gender, as well as clinical data, such as reason for ICU admission, length of ICU stay before BAL, total length of stay at ICU, total length of mechanical ventilation, total length of hospital stay, mortality, alternative pulmonary diagnosis (non-VAP group) and alternative infectious diagnosis (non-VAP group).

### Determination of CC-10 concentration in BAL fluid

CC-10 concentration in the cell-free supernatant of BAL fluid was determined in duplicate by using a commercially available enzyme-linked immunosorbent assay (ELISA) kit (Biovendor Inc., Brno, Czech Republic). The ELISA was performed according to the manufacturer's instructions.

### Quality control of CC-10 concentration in BAL fluid

BAL fluid samples were spiked with a positive control to test for spike recovery.

### Urea concentration analysis

All concentrations of CC-10 were corrected for the dilution factor of the BAL fluid. To compare the concentrations of CC-10 in the BAL fluid samples, the levels were converted to concentrations in the epithelial lining fluid (ELF) by using the urea concentrations in BAL fluid and serum. Therefore, the following formula by Wiedermann *et al.*[26] was used:

[X]ELF = ([X]BAL fluid × urea serum)/urea BAL fluid concentration in which [X] stands for the concentration of CC-10.

In this article, this concentration is referred to as the concentration in BAL fluid. Urea concentrations in serum and BAL fluid were assessed by using a commercially available kit (Urease Method; Beckman Coulter, Fullerton, CA, USA). Urea in both serum and BAL fluid was measured using a Synchron LX20 analyser (Beckman Coulter).

### Statistical analyses

All CC-10 concentrations were logarithmically transformed to obtain normally distributed CC-10 concentrations in the samples. To compare differences in concentrations of CC-10 between the non-VAP and VAP groups, an independent sample *t*-test was used (significance was set at 0.05). For comparison between early- and late-onset VAP, one-way analysis of variance was used (significance was set at 0.05), followed by a Bonferroni *post hoc *test. To ascertain the value of CC-10 in BAL fluid for the diagnosis of VAP, areas under the curve (AUC) of receiver operating characteristic curves were calculated. The statistical analysis was performed using SPSS software version 16.0 for Windows (SPSS, Chicago, IL, USA).

## Results

### Patients included in the study

**Figure 1:**
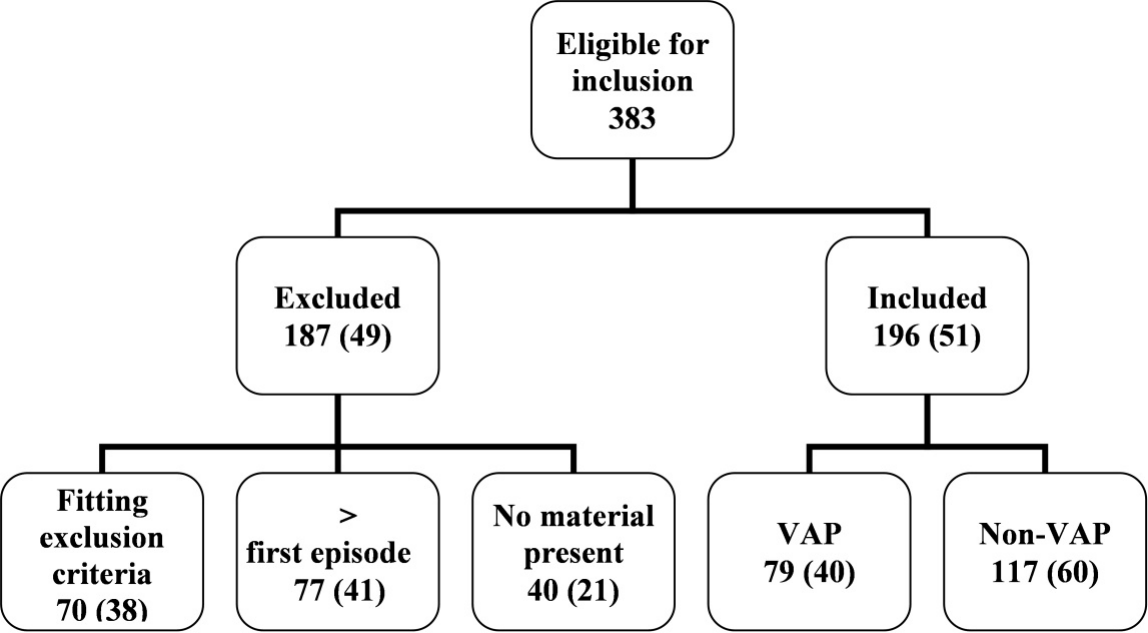
**Inclusion flowchart**. *Percentage between brackets.

Between January 2003 and December 2007, 383 BAL fluid samples were eligible for inclusion in this study. A total of 187 BAL fluid samples were excluded for the following reasons: (1) lack of material (40 BAL fluid samples), (2) not the first episode of suspected VAP in that patient (77 BAL fluid samples), or (3) the fluid sample fitted the exclusion criteria (70 BAL fluid samples). Of the latter 70 samples, 18% were excluded because of poor quality (excessive debris, large percentage of epithelial cells present), 16% were excluded because of a recovered volume < 20 mL and 66% were excluded because of a low total cell count (< 60,000 cells/mL). A total of 196 patients (122 men, 74 women) with a clinical suspicion of VAP were included in the study. Of the 196 episodes of suspected VAP, 79 (40%) were microbiologically confirmed (Figure 1). The patients' characteristics are shown in Table 2. The median age of patients in the VAP group was 64 years (range, 19-84 years) compared with 61 years (range, 18-87 years) in the non-VAP group. Table 3 shows the microorganisms involved in the microbiologically confirmed cases of VAP and in the non-VAP cases. Table 4 shows the alternative pulmonary and infectious diagnoses in the patients included in the VAP group.

**Table 2:** Patient characteristics^a^

Parameter	VAP	Non-VAP	*P *value
Number of patients	79	117	
Mean age in years (range)	60 (19-84)	58 (18-87)	0.445
Male:female ratio	1.6:1	1.7:1	0.887
Mortality rate (%)	44	36	0.324
Median hospital stay in days (range)	47 (7-540)	47 (6-297)	0.289
Median ICU stay in days (range)	43 (6-484)	46 (1-291)	0.454
Median days of intubation (range)	8 (1-172)	8 (1-198)	0.289
Reason for admission, number of patients (%)			
Cardiac	6 (7.6)	12 (10.3)	
Pulmonary	14 (17.8)	27 (23.1)	
Trauma	14 (17.8)	15 (12.8)	
Surgery	17 (21.5)	15 (12.8)	
Neurological	10 (12.6)	6 (5.1)	
Malignancy	5 (6.3)	9 (7.7)	
Vascular surgery	6 (7.6)	19 (16.2)	
Other	7 (8.3)	14 (12.0)	
Median Clara cell protein concentration in ng/mL (range)	3,019 (282-65,546)	2,504 (62-30,240)	0.03

^a^VAP, ventilator-associated pneumonia; ICU, intensive care unit.

**Table 3:** Microorganisms involved in episodes of VAP and non-VAP^a^

Microorganism	VAP n (%)	Early-onset VAP n (%)	Late-onset VAP n (%)	Non-VAP n (%)
*Pseudomonas aeruginosa*	12 (14)	4 (11)	8 (20)	11 (9)
*Staphylococcus aureus*	11 (13)	7 (17)	2 (5)	
*Escherichia coli*	5 (6)	3 (8)	1 (2)	2 (2)
*Proteus *spp.	1 (1)		1 (2)	
*Klebsiella *spp.	7 (9)	6 (16)	2 (5)	
*Stenotrophomonas maltophilia*	2 (3)			
*Moraxella catharrhalis*	1 (1)	1 (3)	2 (5)	
*Serratia *spp.	3 (4)	1 (3)	2 (5)	
*Enterobacter *spp.	3 (4)	1 (3)	2 (5)	
*Haemophilus *spp.	6 (8)	4 (11)		
Mixed	17 (21)	7 (17)	10 (24)	3 (2.5)
Other	11 (13)	4 (11)	7 (17)	
No growth				101 (86.5)
Total (n)	79	38	41	117

^a^VAP, ventilator-associated pneumonia.

**Table 4:** Alternative pulmonary and infectious diagnoses in patients included in the non-VAP group^a^

Alternative diagnoses	Patients n (%)
Alternative pulmonary diagnosis	
Acute respiratory distress syndrome	25 (21)
Congestive heart failure	20 (17)
Diffuse alveolar damage	9 (8)
Idiopathic pulmonary fibrosis	5 (4)
Autoimmune disease	4 (3)
Pulmonary contusion	3 (2.5)
Pulmonary oedema of unknown origin	3 (2.5)
Eosinophilic pneumonia	2 (1.5)
Pneumocystis pneumonia	2 (1.5)
Bronchiolitis obliterans with organizing pneumonia	1 (1)
Drug-induced pneumonia	1 (1)
Chronic obstructive pulmonary disease	1 (1)
Sarcoidosis	1 (1)
*Aspergillus fumigatus *infection	1 (1)
*Legionella pneumophila *infection	1 (1)
No diagnosis	16 (14)
No pulmonary disease	22 (19)
Total (n)	117
Alternative infectious diagnosis	
Intravenous catheter-related infection	7 (6)
Urosepsis	5 (4)
Peritonitis	2 (1.5)
Mediastinitis	2 (1.5)
Encephalitis	1 (1)
Abdominal abscess	1 (1)
No infectious focus found	99 (85)
Total (n)	117

^a^VAP, ventilator-associated pneumonia.

### Spiking recovery of CC-10 in BAL fluid

BAL fluid samples were spiked with different amounts of CC-10. The recovery of the spike reached 92%. Both the low and high concentrations of spiked CC-10 had the highest recovery rates.

### CC-10 concentration in VAP group versus non-VAP group

**Figure 2:**
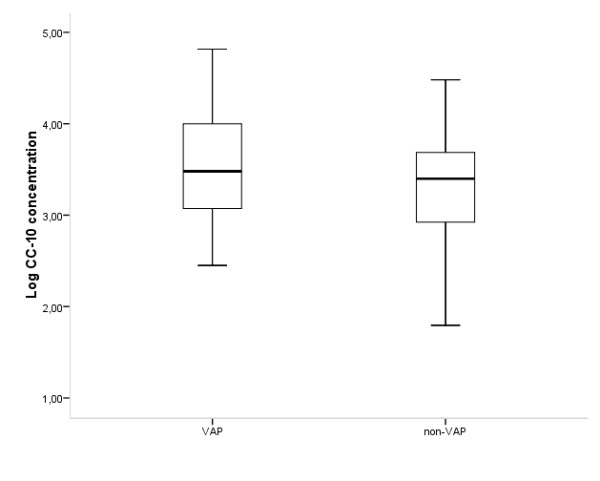
**Comparison of the Clara cell protein concentration between the ventilator-associated pneumonia (VAP) and the non-VAP groups**. Concentrations are given on a logarithmic scale.

**Figure 3:**
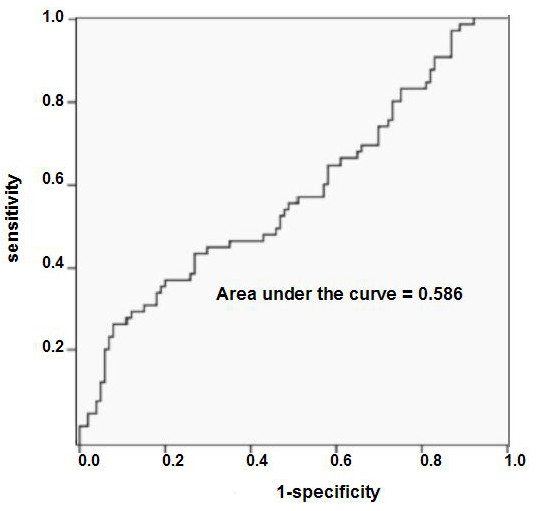
**Receiver operating characteristic for the Clara cell protein concentration**. *P *value, 0.06; 95% confidence interval, 0.496-0.679.

The median CC-10 concentration of the VAP group was 3,019 ng/mL (range, 282-65,546 ng/mL) versus 2,054 ng/mL (range, 62-30,240 ng/mL) in the non-VAP group (*P *= 0.03; 95% confidence interval (95% CI), 0.025-0.380) (Figure 2), with an AUC of 0.586 (*P *= 0.06; 95% CI, 0.496-0.676) (Figure 3). Therefore, the CC-10 levels were not discriminative for VAP. All analyses were also conducted using the uncorrected CC-10 concentrations. However, after logarithmic transformation, these concentrations remained non-normally distributed. For this reason, a Mann-Whitney *U *test was used, which resulted in a *P *value of 0.254 (95% CI, 0.461-0.638]) (data not shown).

### CC-10 concentration in early- and late-onset VAP

The CC-10 concentration between early- and late-onset VAP showed no statistical significance. However, when the non-VAP group was compared with the late-onset VAP group, a significant difference was observed (*P *= 0.04), with an AUC of 0.62 (*P *= 0.29; 95% CI, 0.518-0.731). When the non-VAP group was further divided on the basis of the days of intubation before BAL, no significant difference was observed between the late-onset VAP group and the non-VAP group intubated for more than 7 days (*P *= 0.171; 95% CI, -0.734-0.402). However, a significant difference could be detected between patients with late-onset VAP and non-VAP patients intubated for less than 7 days before BAL (*P *= 0.04; 95% CI, 0.014-0.544).

### CC-10 concentrations in the VAP subgroups versus the non-VAP group

On the basis of the previously described results, the VAP group was subdivided based on the causative organism. Dividing the VAP group into Gram-positive (median, 3.238; and interquartile range (IQR), 0.786) and Gram-negative (median, 3.529; IQR, 1.007) causative organisms yielded no significant result (*P *= 0.06).

**Figure 4:**
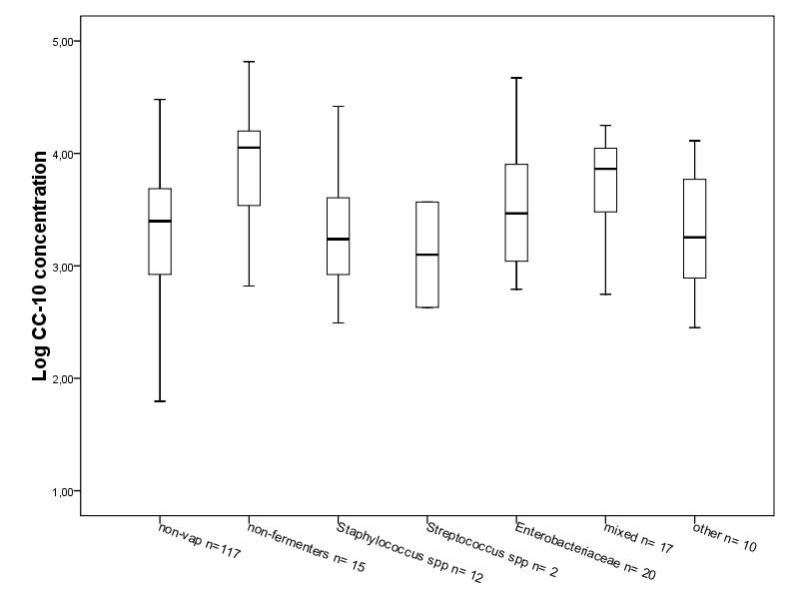
**Comparison of the Clara cell protein concentration between the non-VAP group and the VAP group divided on the basis of the causative organism group**. Concentrations are given on a logarithmic scale.

Analysis of the VAP group was also performed using the following classification of causative organisms found: nonfermenters (for example, *P. aeruginosa, Acinetobacter *spp.), *Staphylococcus *spp., *Streptococcus *spp., Enterobacteriaceae (for example, *Escherichia coli, Klebsiella *spp., *Proteus *spp.), a group in which BAL fluid analysis yielded multiple microorganisms and a group of other causative organisms (for example, *Candida *spp., *Haemophilus *spp.). No significant differences in CC-10 concentrations between the different groups and the non-VAP group (*P *= 0.26) were found (Figure 4).

### Influence of ICU admittance indication on CC-10 concentration

The CC-10 concentrations were compared between the VAP and non-VAP groups on the basis of the category of diagnosis made on ICU admittance: cardiac, pulmonary, traumatic, surgical, neurological and other. No significant differences were observed between the VAP and non-VAP groups (Table 1).

### Antibiotic and corticosteroid therapy at the time of BAL

At the time of BAL, there was no significant difference in CC-10 concentrations between patients with or without corticosteroid treatment (*P *= 0.256; 95% CI, -0.488-0.131) or between patients with or without antibiotic therapy (*P *= 0.909; 95% CI, -0.192-0.215) (data not shown).

## Discussion

The present study shows no correlation between the concentration of CC-10 in BAL fluid and the presence of VAP. Furthermore, CC-10 levels in BAL fluid were not associated with the isolated microorganism.

Previous studies showed that CC-10 concentration in either serum or BAL fluid may be increased in some patients with pulmonary inflammation, for example, due to exposure to lung irritants such as smoke from open fires [27], as well as in patients with acute lung injury and patients with pulmonary fibrosis or sarcoidosis [28].

In contrast to these findings, other types of pulmonary inflammation, such as in patients who have had chronic exposure to tobacco smoke [29,30], as well as in lung transplant recipients with bronchiolitis obliterans and airway neutrophilia [31], have been associated with decreased CC-10 concentration. A study of acute lung injury induced by lipopolysaccharides in rats showed alterations in CC-10 cells [32], which suggests an involvement of CC-10 cells in the inflammatory process induced by bacterial pulmonary infection.

Ye *et al.*[4] measured the concentration of CC-10 in the sera of patients with a variety of pulmonary diseases, including community-acquired pneumonia (CAP). These authors revealed a high CC-10 concentration in patients with IIP and a low CC-10 concentration in patients with CEP and bronchial asthma. However, in patients with sarcoidosis, COPD and CAP, no differences in CC-10 concentration compared with healthy controls were found. Unfortunately, the concentration of CC-10 was measured in serum instead of BAL fluid, and a limited number of patients were included (CAP, *n* = 9; CEP, *n* = 6; IIP, *n* = 11; COPD, *n* = 13; sarcoidosis, *n* = 22).

To the best of our knowledge, the present study is the first in which the value of CC-10 concentration in BAL fluid as a potential marker for VAP has been evaluated. In the present study, the CC-10 concentration was not a useful marker for differentiating VAP from non-VAP, regardless of the type of microorganism causing the patient's pneumonia or the reason for hospitalisation. However, the CC-10 concentration was useful in distinguishing late-onset VAP from non-VAP. A number of possible explanations should be considered. First of all, the type of microorganisms associated with late-onset VAP may be influential. One of the microorganisms frequently associated with late-onset VAP is *P. aeruginosa *[13,25,33]. *P. aeruginosa *is known to produce numerous virulence factors which can destroy the host defence mechanism and facilitate lung infection [25,34]. Harrod *et al.*[5] and Hayashida *et al.*[6], found a decrease in CC-10 expression in cases of *P. aeruginosa *pulmonary infection. Interestingly, the present study did not show a difference in CC-10 concentration when the infection was caused by *P. aeruginosa*. However, the other studies mentioned were based on mouse model experiments [5,6], whilst the present study included ICU patients. Since Clara cell size, mitochondrial morphology, distribution of endoplasmic reticulum and number of Clara cells present in the lung vary between species [35-37], results derived by using mouse models may vary from results derived from studies in humans. By dividing the VAP group into different subgroups on the basis of the causative organism, the number of patients belonging to each group was relatively small. The number of patients with VAP caused by *P. aeruginosa *in the present study may thus be too small to reach statistical significance. A tendency towards significance was observed when the VAP group was subdivided into Gram-positive and Gram-negative causative organisms and compared with the non-VAP group. CC-10 levels were slightly higher in the BAL fluid samples of patients with confirmed Gram-negative VAP. Since Gram-negative microorganisms (especially *P. aeruginosa*) are the major cause of late-onset VAP, the explanations mentioned in the previous section may also be attributed to this tendency towards significance. The second explanation for the fact that CC-10 concentrations distinguished late-onset VAP from non-VAP may be the duration of mechanical ventilation. Dhanireddy *et al.*[38] found that the combination of mechanical ventilation and bacterial infection resulted in increased pulmonary and systemic inflammation. Mechanical ventilation itself may at least partly be responsible for an increase in CC-10 concentrations in all intubated patients. We hypothesise that the difference in BAL CC-10 concentrations found in this study between patients with late-onset VAP and non-VAP may be attributable to the combination of infection and prolonged (> 7 days) mechanical ventilation. This hypothesis is supported by the fact that there was a significant difference between CC-10 concentration in patients in the non-VAP group who had been intubated for less than 7 days and the patients in the late-onset VAP group. However, there was no significant difference between the early-onset VAP group and the non-VAP group intubated for more than 7 days; thus the difference in CC-10 concentration cannot be attributed to the intubation time alone. It is possible that other factors related to BAL fluid influence the recovery of CC-10 levels, since the recovery of the spike was not 100%. However, this would be the case for all BAL fluids analysed in this study.

Because of the retrospective nature of the present study, it was not possible to measure the CC-10 BAL levels during the patients' stay at the ICU. The latter factor may be of interest because some previously investigated proteins, such as procalcitonin, did not show differences when tested once, whilst they appeared to be promising factors in distinguishing between infection and inflammation when tested daily [17,39]. Another limitation of the retrospective nature of this study is that it was not possible to analyse the potential effect of new antibiotics administered to the patients. However, previous studies have shown that neither antibiotics nor corticosteroids influence the concentration of CC-10 [40,41].

## Conclusions

In this study, the CC-10 concentration in BAL fluid was not a useful predictive parameter for the diagnosis of VAP. However, it may be an indicator for pulmonary inflammation in general.

## Key messages

• The CC-10 concentration in BAL fluid is not a useful predictive parameter for the diagnosis of VAP.

• The CC-10 concentration in BAL fluid may be an indicator for pulmonary inflammation in general.

## Abbreviations

AUC: area under the curve; BAL: broncholaveolar lavage; CAP: community-acquired pneumonia; CC-10: Clara cell protein 10; CFU: colony-forming units; CI: confidence interval; COPD: chronic obstructive pulmonary disease; ELF: epithelial lining fluid; ELISA: enzyme-linked immunosorbent assay; ICOs: intracellular organisms; ICU: intensive care unit; IIP: idiopathic interstitial pneumonia; IQR: interquartile range; sTREM-1: soluble triggering receptor expressed on myeloid cells; VAP: ventilator-associated pneumonia.

## Competing interests

The authors declare that they have no competing interests.

## Authors' contributions

MV, CL, JJ, DB and WvM participated in the study design. MV and CL performed the study. MV, CL, JJ and WvM processed the data and performed the statistical analysis. MV, CL and WvM wrote the manuscript. CB, MD, JJ and DB participated in correcting the manuscript. All authors approved the final manuscript.
